# Interfacial Characterization and Thermal Conductivity of Diamond/Cu Composites Prepared by Liquid-Solid Separation Technique

**DOI:** 10.3390/nano13050878

**Published:** 2023-02-26

**Authors:** Yaqiang Li, Hongyu Zhou, Chunjing Wu, Zheng Yin, Chang Liu, Junyou Liu, Zhongliang Shi

**Affiliations:** 1 Institute for Advanced Materials and Technology, University of Science and Technology Beijing, Beijing 100083, China; 2 National Center for Materials Service Safety, University of Science and Technology Beijing, Beijing 100083, China; 3 School of Materials Science and Engineering, University of Science and Technology Beijing, Beijing 100083, China; 4 Qingdao Tianhe Manufacturing Transformation and Upgrading Research Institute Co., Ltd., Qingdao 266400, China; 5 School of Materials Science and Engineering, Baise University, Baise 533000, China

**Keywords:** diamond/Cu composite, thermal conductivity, surface modification, liquid-solid separation

## Abstract

Diamond/Cu composites are widely studied as a new generation of thermal management materials in the field of electronic packaging and heat sink materials. The surface modification of diamond can improve interfacial bonding between the diamond and Cu matrix. The Ti-coated diamond/Cu composites are prepared via an independently developed liquid-solid separation (LSS) technology. It is worth noting that there are obvious differences for the surface roughness between the diamond-{100} and -{111} face by AFM analysis, which may be related to the surface energy of different facets. In this work, the formation of titanium carbide (TiC) phase makes up the chemical incompatibility between the diamond and copper, and the thermal conductivities of 40 vol.% Ti-coated diamond/Cu composites can be improved to reach 457.22 W·m^−1^·K^−1^. The results estimated by the differential effective medium (DEM) model illustrate that the thermal conductivity for 40 vol.% Ti-coated diamond/Cu composites show a dramatic decline with increasing TiC layer thickness, giving a critical value of ~260 nm.

## 1. Introduction

With the rapid development of 5G communication technology, microelectronic products are developing towards high integration, high performance, high speed and miniaturization [1,2,3]. It is necessary to improve the heat dissipation performance of the system to avoid the thermal fatigue failure caused by thermal stress. Diamond particles have become the focus of research on the second-phase particles of electronic packaging materials because of their high thermal conductivity (up to 2000 W·m^−1^·K^−1^). Copper has excellent thermal conductivity, thermophysical properties and processing properties. Therefore, diamond/Cu composites as fourth-generation electronic packaging materials are widely studied because of their outstanding thermal conductivity (TC) and tailorable coefficient of thermal expansion (CTE) [4,5,6]. Additionally, the diamond/Cu composites also have good heat resistance, corrosion resistance, and chemical stability, and they can meet the requirements of extreme service conditions such as high temperature and corrosive environments, such as acid and alkali, as well as complex outdoor atmospheric environments. However, the contact angle between diamond and liquid copper reaches as high as 132.2° even at 1673 K, so the interfacial bonding strength of the two phases is weak [7]. According to the Acoustic Mismatch Model (AMM model) [8,9], the intrinsic physical properties including the density and the phonon velocity of phases on both sides of the interface determine the acoustic impedance and the carrier transfer efficiency. Moreover, the significant difference between diamond and Cu in phonon velocity is not conducive to obtaining high thermal conductivity. Thus, an interfacial modification is of great necessity for diamond/Cu composites. Diamond surface modification with elements such as B, Ti, Cr, and Cu, as a vital method to improve the interfacial bonding between the diamond and the Cu matrix, has attracted much attention in the research of interface modification of diamond/Cu composites [10,11,12,13,14,15]. The effect of Ti-coated on diamond surface is carried out to bring the interfacial TiC layer between the copper matrix and the diamond reinforcements during the sintering process. Furthermore, Ti dissolved with copper at elevated sintering temperatures can form intermetallic compounds with copper between copper and carbide. Moreover, Ti coatings on the diamond surface can improve the wettability between diamond and liquid copper efficiently, owing to the interfacial bonding of Diamond/Cu composites changing from physical bonding or non-bonding to diffusion bonding or chemical bonding. In addition, it can also protect the diamond powder from the atmosphere and reduce the degree of graphitization at high temperatures [16,17]. Because the layer thickness ranges from several nanometers to several microns, an appropriate interlayer as a bridge between the diamond and Cu matrix can effectively reduce the interfacial thermal resistance and thus increase the thermal conductivity of diamond/Cu composites [18].

In the past few years, diamond/Cu composites have mainly been fabricated by hot press sintering [19,20,21], liquid phase infiltration [22,23,24], spark plasma sintering [25,26,27,28] and so on. Although both hot press sintering and spark plasma sintering technologies could be viable routes to reduce cycle time at a low sintering temperature, the fabricated shape of diamond/Cu composites is usually simple. Furthermore, prepared high-volume fraction reinforced diamond/copper composites have low compactness, unstable thermal conductivity, and high requirements for equipment preparation conditions. The pressure infiltration method has the advantages of a short production cycle and fast cooling speed. Still, the equipment required by this technology is complex, and the shape of the prepared composite is small. Pressureless infiltration requires long high-temperature infiltration times, long production cycles, and has low efficiency in large-scale production. Recently, Zhou et al. [29,30,31,32] successfully prepared a liquid-solid separation (LSS) technology for diamond/Al composites, and this method uses a semi-solid metal thixo-forming process, i.e., a solid and liquid two-phase system flown under pressure, which is suitable for maintaining a high-volume fraction of the diamond phase during the preparation of diamond/Al composites. 

In this work, we have attempted to prepare the diamond/Cu composites with some uncoated and a nanoscale layer Ti-coated diamond particles by a liquid-solid separation (LSS) technology for the first time. A mold system was designed to study the liquid phase directional separation control technology, optimize the liquid-solid separation sintering temperature, pressing process, and directional solidification, and prepare high-density Diamond/Cu composites. This method is a compact low-cost processing routine for high-performance diamond/Cu composites. This method has the characteristics of short process flow, near-net forming, simple operation, low requirements for equipment and conditions, and is suitable for large-scale production and industrial application [30]. Moreover, the effects of Ti diamond coatings on the thermal conductivity are demonstrated. Carbon atoms from the diamond and titanium atoms from the coating layer have reacted to form titanium carbide (TiC) during the LSS process, which enhances the interfacial bonding between the diamond particles and the Cu matrix and thus increases the thermal conductivity of the composites. The TC value of 40 vol.% Ti-coated diamond/Cu composite prepared by the LSS technology is 457.22 W·m^−1^·K^−1^, which is 75.3 % of the theoretical value calculated by the Differential Effective Medium (DEM model) of thermal conductivity. The results estimated by the DEM model illustrate that the thermal conductivities for 40 vol.% Ti-coated diamond/Cu composites show a dramatic decline with increasing TiC layer thickness, giving a critical value ~260 nm.

## 2. Experimental Section

### 2.1. Raw Material Preparation

The reinforcement materials were synthetic single-crystalline diamond particles (type HFD-B, average particle size ~102 μm, Henan Huanghe Whirlwind Co., Henan, China). The surfaces of the diamond particles were modified and deposited with a nanoscale Ti layer by a vacuum ion plating process, and the thickness of the coating layer was approximately 100 nm. Furthermore, commercially available bulk spherical copper powder (purity 99.85 wt.%, average particle size ~45 μm, Zhongnuo New Material Beijing Technology Co., Ltd., Beijing, China) was used as metal matrix. The nitrogen content in these diamond particles was characterized to be (190~200) × 10^−6^ by the inert gas fusion-infrared TC method and thus the corresponding TC was calculated at about 1450 W·m^−1^·K^−1^ [33,34].

### 2.2. Composite Preparation

**Figure 1:**
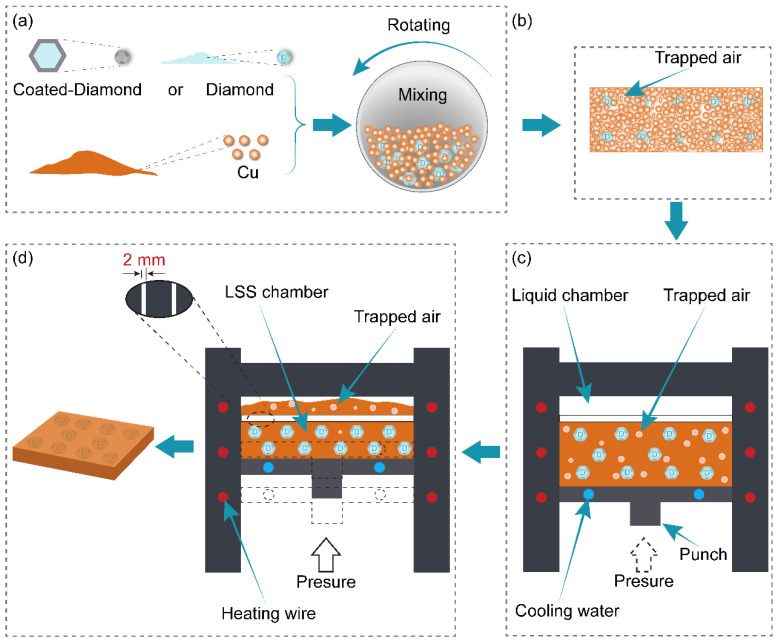
(**a**–**d**) Schematic diagram of the liquid-solid separation (LSS) technology.

As shown in Figure 1, the diamond/Cu composites containing 40 vol.% of diamond were fabricated by a liquid-solid separation (LSS) technology route using the copper powder and the diamond particles. The detailed preparation methods for the composites are described below. Firstly, as shown in Figure 1a, the diamond particles and copper powder were mechanically mixed with the volume ratio of 1:4 in the omni-directional planetary mill (type LGB2, Nanjing Nanda Instrument Co., Ltd., Nanjing, China) for 5 h. Secondly, the mechanically mixed powder was compressed into a billet by a Four-column hydraulic press (YQ28−100, Wodda, Shandong, China) at 300 MPa for 2 min, as shown in Figure 1b. Thirdly, as illustrated in Figure 1c, the cold-pressed billet was heated with a heating rate of 15 K·min^−1^ from room temperature to 1376 K in the sintering stage, at which the temperature was held for 30 min to make the billet reach the liquid-solid mixed state. Fourthly, as shown in Figure 1d, the liquid-solid mixed melt slurry was squeezed with the piston at a pressure of 100 MPa. The assumed volume of copper liquid in the liquid-solid mixed melt slurry was squeezed into the liquid chamber through the 2 mm liquid-solid separation channel. At the same time, the diamond particles were utterly retained in the LSS chamber. Immediately, the remaining liquid-solid mixed slurry was solidified layer-by−layer from bottom to top under the water-cooling system maintained with the continuous pressure holding for 10 min. These diamond/Cu composite samples have a cuboid shape with a size of 50 mm × 40 mm × 3 mm.

### 2.3. Characterization

An atomic force microscope (AFM, Bruker Dimension Icon, Salbuluken, Germany) was used to characterize the 3D topography and roughness of raw and Ti-coated diamond particles. The diamond/Cu composites were machined to a particular size via laser cutting and a diamond grinding wheel. In order to investigate the interfacial bonding of the coating layer on the diamond surface, an argon ion beam cross section polisher (IB−19530CP, JEOL, Tokyo, Japan) was applied to prepare the samples of interfacial microstructure. The surface microstructure and interface morphology of the composites were characterized via field emission scanning electron microscopy (SEM, SUPRA 55, ZEISS, Oberkochen, Germany) equipped with energy-dispersive spectroscopy (EDS). The distributions of C, Ti, and Cu elements between Cu and diamond particles were detected using a JXA−8230 electron microprobe analysis (EMPA, JEOL, Japan). The phase composition of the Ti-coated diamond particles and composites was characterized by Advance D8 X-ray diffraction (XRD, Bruker, Germany) with Cu Kα radiation. XRD patterns were performed in the 2 ranges from 20° to 100° with a scan speed of 5°·min^−1^. The surface element composition and chemical structure transformation of the diamond particles extracted from the composites were detected by X-ray photoelectron spectroscopy (XPS, K-Alpha, Thermo Scientific, Waltham, MA, USA) using a monochromatic Al Κα X-ray source. The thermal diffusivity (*α*) was tested on a laser scattering TC instrument (LFA 427, NETZSCH, Free State of Bavaria, Germany) at room temperature with a sample size of φ 12.7 mm × 3 mm. The TC was calculated as a product of the thermal diffusivity (*α*), the density (*ρ*), and the specific heat capacity (*C_p_*). The densities of the composites were measured via Archimedes using distilled water as the immersion medium, and the specific heat capacity (*C_p_*) at constant pressure was calculated via the rule of mixture (ROM) according to the mass fraction of each component. The coefficient of thermal expansion (CTE) of the composites was measured by a DIL 402C thermomechanical analyzer (NETZSCH, Free State of Bavaria, Germany) with cuboid-shaped specimens of 4 mm × 3 mm × 25 mm at a heating rate of 5 K·min^−1^ from room temperature to 623 K. Three or more specimens were calculated for each measurement to reduce system error.

## 3. Results and Discussion

### 3.1. Analysis of Microstructure and Morphology of Diamond Particles

**Figure 2:**
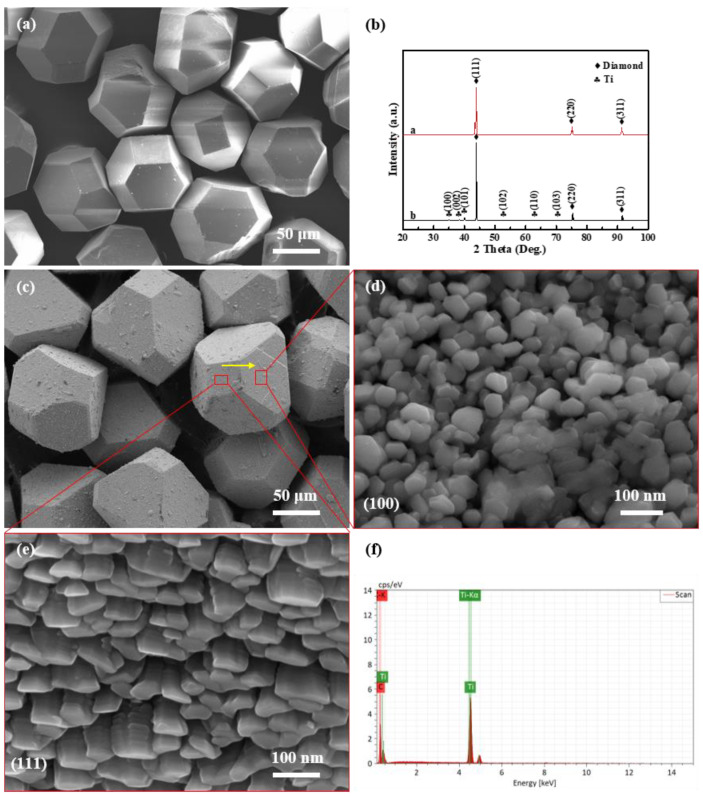
SEM observation and XRD analysis: SEM image of (**a**) raw diamond and (**c**) Ti-coated diamond particles as well as (**d**,**e**) (100)—oriented and (111)—oriented diamond plate, as well as (**b**) XRD patterns of raw diamond particles and Ti-coated diamond particles, as well as (**f**) EDS element line of (**c**).

Figure 2 shows the SEM morphologies of uncoated diamond particles and diamond particles coated with titanium and their XRD patterns. Obviously, the raw diamond particles have flat and smooth surfaces, except for a minor defect shown in Figure 2a. The surface of Ti-coated diamond particles is coarser than that of raw diamond particles. All of the diamond particles retain their original shape of a complete hexahedron octahedron after deposition. As exhibited in Figure 2d,e, there are two different morphologies of the titanium layer on its surface, where nanoscale particles with a {111} preferred orientation and columnar structures with a {100} preferred orientation are present. The XRD patterns in Figure 2b show the diffraction peaks of diamond and titanium, and no peaks of titanium carbide are detected. The result indicates that titanium will not react with the diamond in vacuum ion plating process at a relatively low temperature [16]. Moreover, the result of the EDS line scanning analysis of titanium and carbon confirms that all of the crystal planes are coated thoroughly, as illustrated in Figure 2f.

**Figure 3:**
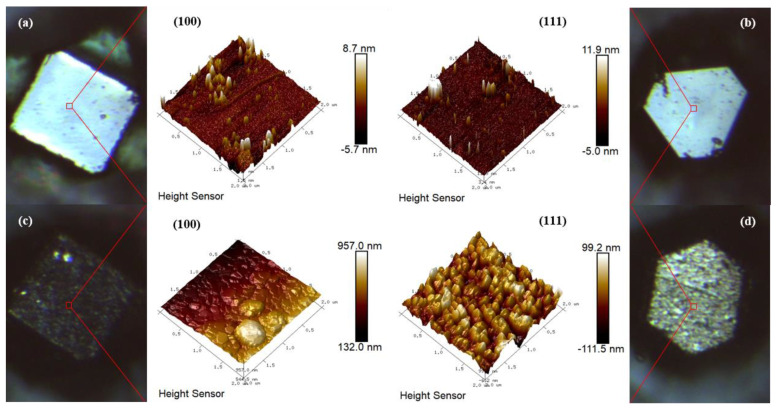
AFM 3D images of diamond surface: (**a**,**b**) raw diamond, (**c**,**d**) Ti-coated diamond.

As shown in Figure 3, AFM 3D images of raw diamond particles and Ti-coated diamond particles are examined to research their surface characteristics further. For the former, as seen in Figure 3a,b, the surfaces on the raw diamond-{100} and -{111} facets are relatively smooth and flat. For the latter, as illustrated in Figure 3c,d, the titanium grain sizes on the diamond-{100} facet are more extensive than those on the diamond-{111} facet, but they are both in nanoscale. Additionally, the result is similar to the SEM images shown in Figure 2c–e. Obviously, the existence of pits and protruding interfaces is conducive to the stable combination between the diamond surface and the matrix copper in the subsequent liquid-solid separation process [35]. In addition, the root mean square (RMS) surface roughness of the Ti-coated diamond-{100} facet is measured to be ~29 nm, higher than that of the diamond-{111} facet (R_a_ = 23.9 nm) by AFM. The anisotropy of the titanium deposition on the diamond surface might be explained by the gas desorption temperature and the surface energy of different facets [36,37]. A larger surface roughness means a more prominent disordered region between the peaks and valleys on the titanium coating surface, since the electron transport at the metal interfaces will be controlled by disordered elastic scattering [38,39]. Therefore, a reasonable thickness of the interfacial coating is essential to the thermal conductivity of composites.

### 3.2. Microstructure and Interfacial Characteristics

**Figure 4:**
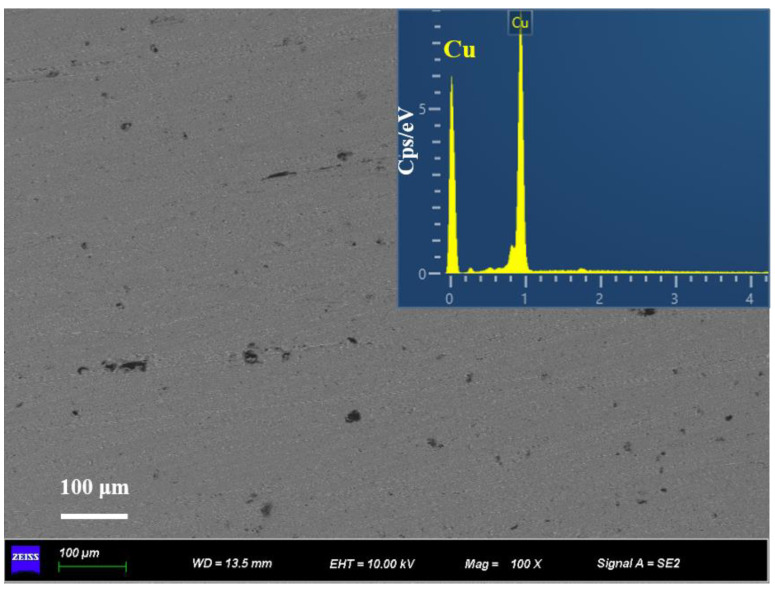
SEM image and EDS element mapping of the squeezed—out liquid phase.

Figure 4 shows the microstructure of the separated liquid phase and the EDS element mapping in the corresponding region, which proves that the field of view is pure copper and no single-crystal diamond particles exist in the separated liquid phase. It means that the 2 mm channel can retain the diamond particles in the LSS chamber. Therefore, the diamond/Cu composites with different volume fractions are precisely fabricated by changing the volume of the LSS chamber. In general, there are some pores among the diamond particles and the copper powder during cold pressing [40]. However, the pores eventually dissolved as the LSS process completed, which is essential for the high relative density of diamond/Cu composites.

**Figure 5:**
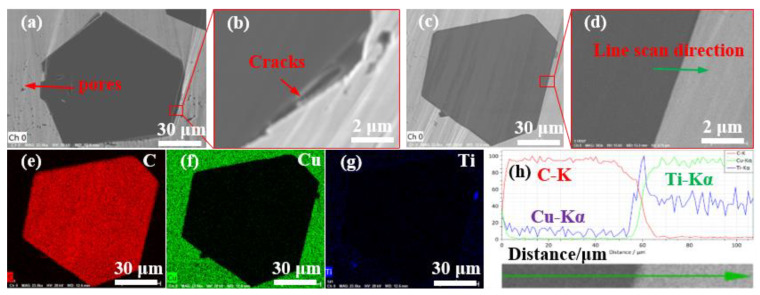
SEM images of the polished surface of (**a**,**b**) 40 vol.% Diamond/Cu composites and (**c**,**d**) 40 vol.% Diamond^Ti^/Cu composites, and (**e**–**g**) elemental mappings of C, Cu, and Ti in (**c**), and (**h**) EDS elemental liner results of the regions in (**d**).

Figure 5 shows 40 vol.% Cu/diamond composites by LSS technology with uncoated diamond particles and Ti-coated diamond particles. It can be observed from Figure 5a,b that there are obvious cracks and pores between the uncoated diamond particle and the Cu matrix. Moreover, these defects are equivalent to air, while the thermal conductivity of air is only 0.026 W m^−1^ K^−1^ [41], which obviously reduces the thermal conductivity of diamond/Cu composites. Moreover, the chemical incompatibility and the different thermal expansion coefficients between the diamond particles and copper matrix are also revealed. Figure 5d shows that the diamond particles are closely bonded to the Cu matrix for the Ti-coated diamond/Cu composites. The EDS results obtained along arrowheads in Figure 5c are depicted in Figure 5h. It is clear that the titanium element is distributed at the interface between diamond and copper, and the elements carbon, copper, and titanium have some mutual diffusion at the interface. The XRD patterns of Ti-coated diamond/Cu composites and the XPS spectrum of diamond particles extracted from them in the subsequent content demonstrate that carbon atoms from the diamond and titanium atoms from the coating layer have reacted to form titanium carbide (TiC) during the LSS process. The possible factors for enhancing interfacial bonding are as follows. On the one hand, the formation of titanium carbide can improve the bonding of the interface between the diamond particles and copper matrix.

On the other hand, the occurrence of an interfacial diffusion reaction between titanium and copper at a high temperature may enhance the interfacial bonding. However, there may be still a rare amount of titanium or Cu (Ti) solid solution to remain in the composites, which is not detectable by XRD due to its low quantity.

**Figure 6:**
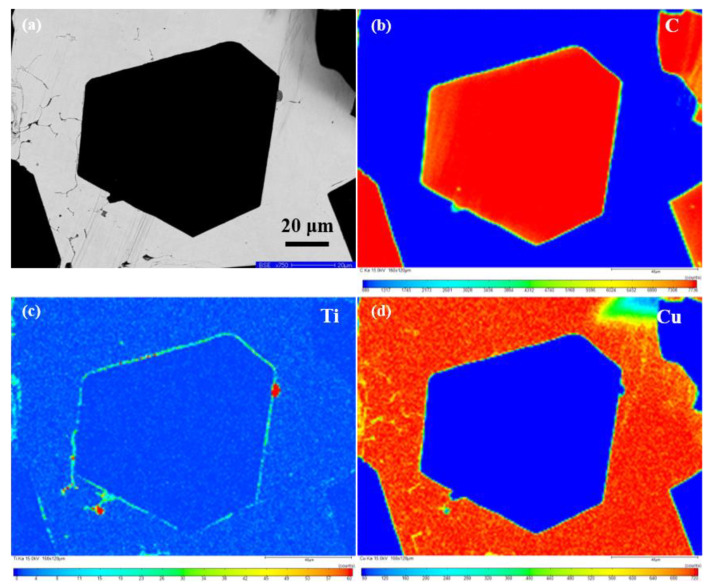
EMPA area—scan of Diamond^Ti^/Cu composite: (**a**) BSE image and (**b**–**d**) elemental distribution mappings of C, Ti and Cu.

Figure 6 presents the element distribution maps on the polished surface of the Ti-coated diamond/Cu composite. The SEM image in the backscattered electron (BSE) mode (Figure 6a) shows a whole diamond particle embedded in the copper matrix used for EMPA characterization. From the inside of the diamond to the boundary of the diamond, has gradually changed from carbon to titanium. It can be seen that titanium enriches along the interface between the diamond and copper matrix and a rare part distributes in the matrix. It is consistent with the results of EDS in Figure 5e–h, which indicates that the transition layer at the interface of the Ti-coated diamond/Cu composite are mainly titanium-containing phases. The results of XRD and XPS confirm that the bonding phases on the interface between the diamond particles and copper matrix are identified as titanium carbide (TiC). Interestingly, the high content in a few positions may be related to the enrichment of titanium on the surface of diamond particles during the vacuum ion plating process. Moreover, it can be assumed that titanium carbide nucleation occurs preferentially at the diamond facets’ surface defects, where carbon may be more easily liberated to form carbide.

**Figure 7:**
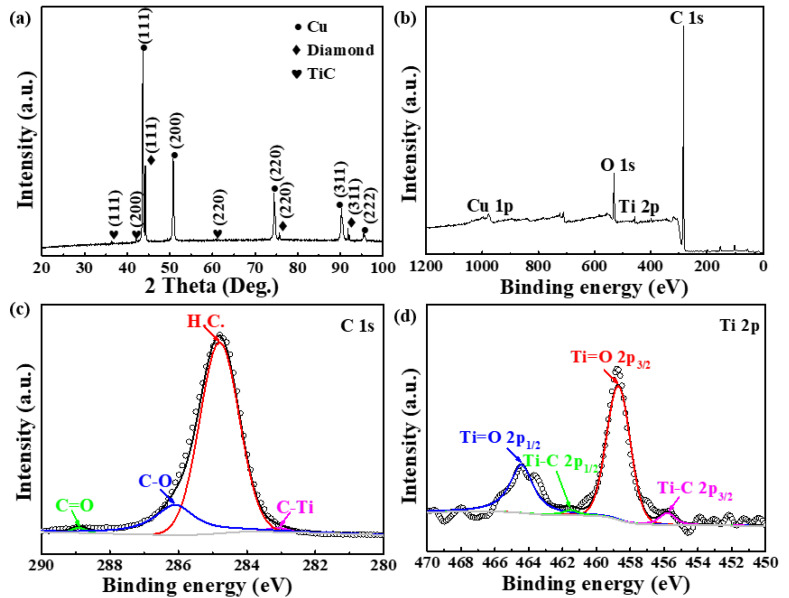
(**a**) XRD patterns of Diamond^Ti^/Cu composites and (**b**) XPS spectrum and (**c**) high—resolution XPS spectra of C1s and (**d**) high—resolution XPS spectra of Ti 2p of the diamond particles extracted from Diamond^Ti^/ Cu composites.

The Gibbs free energy (ΔG) of the formation of titanium carbide is from −174 kJ mol^−1^ to −169 kJ mol^−1^ between 1193 K and 1323 K [42], which is beneficial to the formation of interfacial titanium carbide. Figure 7a shows the XRD pattern of the Ti-coated diamond/Cu composites which displays the weak diffraction peaks corresponding to the (111) _TiC_, (200) _TiC_, and (220) _TiC_, respectively. Therefore, it is thermodynamically possible that a trace of Cu (Ti) intermetallic compounds has been obtained via the reaction between diamond and titanium coating during the LSS process. It has been proved that Ti and Cu have negative values of the Gibbs free energy (ΔG) of mixing and can form a series of intermetallic compounds such as CuTi_2_, CuTi, Cu_3_Ti_2_, Cu_4_Ti_3_, etc., at the temperature below 1213 K [43]. However, the Ti-Cu intermetallic compounds are not detected by XRD. The reason for this is that the Ti-Cu intermetallic with higher Gibbs free energy (ΔG) formed during the LSS process is a metastable phase and reacts with the carbon atom to form titanium carbide with lower Gibbs free energy (ΔG) [44]. On the other hand, the titanium atom preferentially diffuses to the surface of diamond and reacts with the carbon atom to titanium carbide on the diamond surface heterogeneously and then grows preferentially.

To further analyze the chemical composition of the interfacial layer’s surface between the diamond and copper, XPS analysis of the diamond particles extracted from the Ti-coated diamond/Cu composites was conducted, as shown in Figure 7b–d. The XPS analysis reveals the chemical composition containing C, O, Ti, and Cu, as shown in Figure 7b. High-resolution XPS spectra at C 1s in Figure 7c shows that there are four apparent peaks in the C 1s region, and they are attributed to 283.1 eV (C-Ti), 288.9 eV (C=O), 286.1 eV (C-O), and 284.8 eV (H.C.), respectively. Figure 7b displays the Ti 2p XPS spectra and the Ti 2p_3/2_ has been split into two peaks. The weak peaks at 455.8 eV and 461.52 eV are ascribed to the Ti 2p_1/2_ and Ti 2p_3/2_ in TiC. Above these indicate the formation of TiC due to the reaction between diamond and titanium, which conforms with the XRD results. In addition, the oxygenated carbon functional groups are likely from the oxygen in the atmosphere during the LSS process.

### 3.3. Thermal Properties

The phonon is the heat transmission medium of diamond and carbide. However, the electron is the carrier of heat transmission in the matrix copper when heat transmission occurs in the diamond/Cu composites. In addition, the phonon density of states of copper and diamond do not overlap. Thus the phonon scattering caused by the subsurface disorder and imperfect physical contact in the interfaces of diamond/Cu composites limits heat transmission mechanisms at the interface [9,45].

Considering the influence of various factors such as the volume fraction and the particle size of the diamond, the intrinsic thermal conductivity of the diamond and copper, and the interface characteristics on the thermal conductivity of composites, the theoretical thermal conductivity of diamond/Cu composites is expected to be predicted by the DEM theoretical model [46,47,48], as follows:

(1) (λCλm)13(1−Vd)=λdeffλm−λCλmλdeffλm−1

(2) $$\lambda_{d}^{\mathrm{eff}}=\frac{\lambda_{d}^{\mathrm{in}}}{1+\frac{\lambda_{d}^{\mathrm{in}}}{{r\times h}_{C}}}$$

where λ is the thermal conductivity and the subscripts m, d and C denote the copper matrix, diamond, and composites, respectively. Moreover, $\lambda_{d}^{\mathrm{eff}}$ is the intrinsic thermal conductivity of the diamond. *h*, *V*, and *r* and the interfacial thermal conductivity (ITC) of the composites, the volume fraction, and the average radius of the diamond, respectively.

(3) $$h_{C}=0{.25\rho C}_{p}\eta_{1\to 2}v_{D}$$

(4) $$\mathrm{with}\;\eta_{1\to 2}\approx \frac{{2Z}_{1}Z_{2}}{{{(Z}_{1}{+Z}_{2})}^{2}}{\left(\frac{v_{1}}{v_{2}}\right)}^{2}$$

In the Equation (3), ρ and $C_{p}$ refer to the density and heat capacity of phonon emission, respectively. $\eta_{1\to 2}$ is the scattering coefficient of received phonons and the subscripts “1” and “2” represent the emitting phase and receiving phase of phonons, respectively. The Equation (4) can be calculated through the Acoustic Mismatch Model (AMM) [49,50,51], where *Z* and *v* are phonon impedance (Z=ρv) and the speed of sound, respectively.

(5) vD=(13vl3+23vt3)−13

(6) vl=(B+4G3ρ)12

(7) vt=(Gρ)12

$v_{D}$ is Debye model, and the expression is shown in Equation (5), where $v_{l}$ and $v_{t}$ refer to the longitudinal phonon velocity and the transverse phonon velocity through the matrix [52,53]. Moreover, $v_{l}$ and $v_{t}$ can be calculated through the Equations (6) and (7), where *B* and *G* are the bulk modulus and shear modulus of the material, respectively.

(8) $$\frac{1}{h_{C}}=\frac{1}{h_{m/Carbide}}+\frac{l_{Carbide}}{\lambda_{Carbide}}+\frac{1}{h_{Carbide/d}}$$

The total interfacial thermal conductance (*h_c_*) of the Ti-coated diamond/Cu composites can be determined by using the concept of interfacial thermal resistance, as shown in the Equation (8) [54], where $h_{m/Carbide}$ refers to the thermal conductivity of the interface between the copper matrix and the carbide transition layer, $h_{Carbide/d}$ refers to the interfacial thermal conductivity between the diamond and carbide transition layer, and $\lambda_{Carbide}$ and $l_{Carbide}$ refer to the thermal conductivity and thickness of carbide transition layer, respectively.

**Figure 8:**
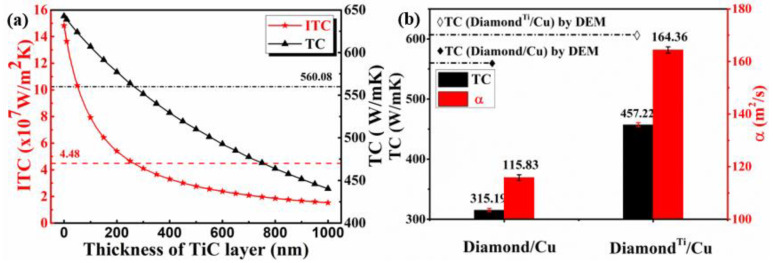
(**a**) ITC and TC of 40 vol.% Diamond^Ti^/Cu composites with TiC layers with different thickness, and (**b**) The TC and α of 40 vol.% Diamond/Cu and Diamond^Ti^/Cu composites with the ~100nm TiC layer.

As shown in Figure 8b, the TC value of 40 vol.% diamond/Cu composites prepared by the LSS technology is only 315.19 W·m^−1^·K^−1^, which is lower than the corresponding DEM theoretical model value (560.08 W·m^−1^·K^−1^). It can be seen from the SEM images in Figure 5b that the cracks and pores between the uncoated diamond particle and Cu matrix enhance the phonon scattering and thus reduce the thermal conductivity. However, the interface between the diamond and copper strengthens when a nano-sized Ti layer coats the surface of the diamond particle through a vacuum ion plating process. Eventually, a well-bonded TiC interface makes up for the high interface thermal resistance due to the difference of acoustic impedance between the diamond and Cu.

The thermophysical parameters of materials used in the calculation of thermal conductivity are listed in Table 1. It can be seen that the thermal conductivity of TiC (17 W·m^−1^·K^−1^ [55]) is much lower than that of diamond (1450 W·m^−1^·K^−1^) or Cu (400 W·m^−1^·K^−1^). Therefore, the value of $\frac{l_{TiC}}{\lambda_{TiC}}$ has a significant influence on the composites’ interfacial thermal resistance, so the interfacial carbide layer should be as thin as possible to minimize the interfacial thermal resistance. In order to simplify the calculation, based on the results of XRD and XPS analysis, only the TiC layer is considered to calculate the values of ITC and TC. The results of DEM predictions for the 40 vol.% uncoated and Ti-coated diamond/Cu composites with different thicknesses of TiC layer are plotted in Figure 8a. It is seen that the derived value of ITC and TC for the composites show a dramatic decline with the TiC layer thickness increasing, and they might be negative when the thickness exceeds a critical value (~260 nm). When the thickness of the TiC layer is 100 nm, the DEM theoretical model of thermal conductivity for the Ti-coated diamond/Cu composite is 607.1 W·m^−1^·K^−1^ in this work. The TC value and relative density of 40 vol.% Ti-coated diamond/Cu composite prepared by the LSS technology are 457.22 W·m^−1^·K^−1^, 98.1% respectively, which are both higher than these of 40 vol.% uncoated diamond/Cu composite. The result exhibits a transition from weak to solid interface bonding when the diamond particles are coated with a nanoscale thickness titanium layer, which plays an influential role in enhancing the interfacial bonding between the diamond and copper matrix.

## 4. Conclusions

(1) The diamond/Cu composites with 40 vol.% uncoated and Ti-coated diamond particles were successfully fabricated via a liquid-solid separation (LSS) technology, respectively. Moreover, the diamond/Cu composites with different volume fractions can be precisely fabricated by changing the volume of the LSS chamber.

(2) The TC value of 40 vol.% Ti-coated diamond/Cu composite reached 457.22 W·m^−1^·K^−1^, which was 75.3 % of the theoretical value. Compared with 40 vol.% uncoated diamond/Cu composite, the thermal conductivity increased by nearly 50%. The high thermal conductivity is attributed to the formation of the TiC between the titanium and diamond at the diamond/Cu interface, which improves the interfacial bonding between the Cu matrix and diamond particles.

(3) We have analyzed that the root mean square surface roughness of the Ti-coated diamond-{100} facet was measured to be ~29 nm, higher than that of the diamond-{111} facet (R_a_ = 23.9 nm) by AFM. Due to the different surface roughness, the electron transmission among the Cu interface will be disordered elastic scattering. Therefore, a reasonable thickness of the interfacial coating is essential to the thermal conductivity of composites. The nano-thickness titanium layer significantly influences the thermal conductivity of the interface between Cu and diamond on the composites.

(4) In this work, the Ti—Cu intermetallic compounds were not detected. When the thickness of the TiC layer is 100 nm, the DEM theoretical model of thermal conductivity for the Ti-coated diamond/Cu composite is 607.1 W·m^−1^·K^−1^. The results estimated by the differential effective medium (DEM) model illustrate that the thermal conductivities for 40 vol.% Ti-coated diamond/Cu composites show a dramatic decline with increasing TiC layer thickness, giving a critical value ~260 nm.

## Figures and Tables

**Table 1:** Parameters and calculated values of materials used in the calculation of thermal conductivity [56].

Materials	*C_p_*/ J·Kg^−1^·k^−1^	TC/ W·m^−1^·K^−1^	*ρ*/ Kg·m^−3^	*V_l_*/ m·s^−1^	*V_t_*/ m·s^−1^	*V_D_*/ m·s^−1^	*B*/ GPa	*G*/ GPa	h × 10^8^/ W·m^−2^·K^−1^
Diamond	512	1450	3520	17,500	12,800	13,805	—	—	h_Cu/d_ = 4.48
Cu	386	400	8960	4910	2500	2801	—	—	h_Ti/d_ = 0.62
TiC	569	17	4930	—	—	6777	240	186	h_TiC/d_ = 5.53
Ti	522	22	4540	—	—	3730	—	—	h_Cu/TiC_ = 2.03

## Data Availability

The data are available from the corresponding author on reasonable request.
